# Supplementary material for: A nonsense mutation in myelin protein zero causes congenital hypomyelination neuropathy through altered P0 membrane targeting and gain of abnormal function
Source: Hum Mol Genet. 2018 Sep 19;28(1):124–32. doi: 10.1093/hmg/ddy336 (PMC6298235; doi:10.1093/hmg/ddy336)
Supplement: Supplementary Data [file ddy336_supp.docx]

**Material and Methods**

***Production of Targeting Vector***

The complete *Mpz* was cloned from a a-DASH II mouse (129SV strain) genomic DNA library, a generous gift from Andras Nagy (Mount Sinai Hospital, Toronto, Canada), and subcloned into pBluescript SKII (Stratagene, La Jolla, USA), in order to obtain the *Mpz*blue plasmid. To generate the Q215X targeting vector, a 2.6-kb BsrGI fragment, containing exons 2, 3, 4 and 5 was excised from Mpzblue, blunted and subcloned into blunt pBluescript (*EcoRI* digested and subsequently filled-in), to generate the plasmid SA_wt_blue. Polymerase chain reaction (PCR)-based, site-directed mutagenesis (Ho et al., 1989) was performed on a NcoI-EcoRV fragment of SA_wt_blue, in order to introduce the Q215X point mutation in exon 5. The 2.6-kb *SmaI-EcoRV* mutated fragment was excised from the resulting plasmid (SA_Q215X_blue) and subcloned into a SmaI digested pLOX plasmid (kind gift of Dr. P Orban), 5’ to a floxed neo selection minigene present in pLOX, to obtain the plasmid SA_Q215X_LOX. A 4-kb *BsrGI-SalI* fragment was excised from *Mpz*blue and subcloned 3’ to the floxed neo cassette, into SalI-BsrGI digested SA_Q215X_LOX, in order to generate a new plasmid termed SA+1/2LA_Q215X_LOX. Finally, to obtain the complete Targeting Vector, to be used for homologous recombination in ES cells, a 4-kb BsrGI fragment was excised from *Mpz*blue and subcloned into BsrGI digested SA+1/2LA_Q215X_LOX plasmid. The resulting TV_Q215X_ plasmid was linearized using *NotI* endonuclease and electroporated into ES cells. In order to construct the TV_wt_ for the generation of the control LoxP mouse, we used the same strategy, except that the 2.6 BsrGI fragment was not mutated.

***Generation of recombinant embryonic stem (ES) cells***

ES cells (TBV2 line - Corradi, 2003) were grown in Dulbecco's Modified Eagles Medium (DMEM) 15% fetal calf serum, 10^-4^ M ß-mercaptoethanol (all from GIBCO Industries, Langley, OK, US), 2 mM L-Glutamine and 10^7^ U/ml LIF (CHEMICON Int.- Temecula, CA, US), on an embryonic fibroblast feeder layer previously inactivated with Mitomycin C. Electroporation and positive selection were performed and resistant colonies were picked after 8-10 days of selection. Genomic DNA was extracted from expanded clones, digested with HindIII and analysed by Southern blotting at the 5' end of the recombinant locus. Homologous recombinant clones were analysed at the 3' end by HindIII and BglII digestion and Southern analysis. Out of 168 ES clones screened, one scored positive for Q215XNEO homologous recombination and was propagated. 2 clones, out of 190, scored positive for LoxP homologous recombination and were propagated. The Q215XNEO positive clone was screened for the presence of the mutation by PCR analysis, using intron 4 and intron 5 specific primers (5’-CCCTAGACTGCTTCAGTGGTGG-3’ and 5’-GGTCAGCCTTGGGCTTGAC-3’ respectively), followed by restriction with BsoFI endonuclease. PCR conditions were 95°C for 30s, 55°C for 60s and 72°C for 30s (30 cycles), followed by 10 min extension at 72°C, in a standard PCR reaction mix.

***Generation of chimeric mice and germline transmission of the Mpz targeted allele***

All experiments performed on mice were conducted with appropriate anaesthesia, in accordance with experimental protocols approved by the Institutional Animal Care and Use Committee, San Raffaele Scientific Institute and the Italian ministry of Health. The targeted ES clones were injected into blastocysts derived from C57BL/6J females. The chimeric embryos were then transferred into the uteri of 2.5-day pseudopregnant foster mothers. Chimeric males with 70-100% agouti colour were bred by crossing with wild-type C57BL/6J females and germline transmission was identified by the presence of agouti offspring. Genotyping was carried out by multiplex PCR amplification with one primer in intron 4, another one in exon 6 and the third one on the neomycin minigene (respectively: 5’-CCCTAGACTGCTTCAGTGGTGG-3’, 5’-GGTGCTTCGGCTGTGGTCC-3’ and 5’-CAATGACGACGCTGGGCGGGG-3’). PCR conditions were 95°C for 60s, 68°C for 60s and 72°C for 120s (32 cycles), followed by 10 min extension at 72°C, in a standard PCR reaction mix. Heterozygous mice for the Q215XNEO allele were subsequently confirmed by Southern blotting using the 5' probe. In order to remove the neomycyn minigene, we then crossed Q215XNEO heterozygous mice with transgenic mice expressing the Cre recombinase under control of the human cytomegalovirus minimal promoter (Schwenk et al., 1995). We obtained neo negative, Q215X heterozygous mice, that were then crossed into homozygousity. Genotyping of these mice was carried out by PCR using intron 4 and exon 6 specific primers. Both Southern blot and PCR analyses were performed on genomic DNA prepared from tail samples. Q215X heterozygous mice were then maintained by backcrosses to FVB/N mice (Charles River Lab.). Animals used for most experiments were N5 to N11 in FVB/N background.

***Transgenic mouse line***

All experiments involving animals were performed in strict accord with experimental protocols approved by the San Raffaele Scientific Institute Animal Care and Use Committee. *Mpz^Q215X/+^* and *Mpz^Q215X/Q215X^* knock-in mice, *Mpz^+/+^, Mpz^+/-^* and *Mpz^-/-^,* P0 overexpressing mice (*Mpz^+/+;tgP0oe^*), *Mpz^+/+;Myc^* and P0-S63del transgenic mice were maintained on the FVB/N (Charles River, Calco, Italy) genetic background; *Mpz^LoxP/LoxP^* knock-in mice were maintained in C57B6/N genetic background. Genotype analysis of *Mpz^+/+;tgP0oe^*, P0-S63del and *Mpz^-/-^* mice was performed as described previously (Wrabetz et al, 2000; Wrabetz et al, 2006; Giese et al, 1992). Genotype of *Mpz^Q215X/Q215X;tgP0oe^* mice was determined with the 186 PCR and genotype of *Mpz^+/+;Myc^* mice was determined with the 140 PCR for the presence of the transgene 140 (myc tag at the C terminal of P0; Fratta et al, 2011). Genotypes of *Mpz^Q215X/+^*, *Mpz^Q215X/Q215X^* and *Mpz^LoxP/LoxP^* knock-in mice were determined with the 186 PCR. *186 PCR:* primer 186S: CAACCTCTCTTGCCACAGTG, primer 186AS: GCTAACCGCTATTTCTTATCC; *140 PCR:* primer 186S: CAACCTCTCTTGCCACAGTG, primer 140AS: CAAGTCCTCTTCAGAAATGAGC

***Western Blot Analysis***

Frozen sciatic nerves dissected from P28 Q215X heterozygous, homozygous and wild type mice were pulverized, sonicated in lysis buffer (95 mM NaCl, 25 mM Tris-HCl, pH 7.4, 10 mM EDTA, 2% SDS, and protease inhibitors), boiled for 5 min, and spun at 14,000 rpm in a microcentrifuge for 10 min at room temperature to eliminate insoluble material. The protein concentration in supernatants was determined by BioRad protein assay according to the manufacturer's instructions. Equal amounts of homogenates (containing 2.5–10 µg of protein) were brought up to 5 µl with 8 M urea, to which was added 5 µl of 8 M urea, 0.05 M DTT, 1%SDS, followed by 10 µl of standard reducing sample buffer. The samples were denatured, resolved on a 14% SDS-polyacrylamide gel, and electroblotted onto PVDF membrane. To verify equal loading of protein, membranes were stained with amido black or ponceau red. Blots were then blocked with 0.05% Tween, 5% dry milk in PBS, and incubated with the appropriate antibody in 0.05% Tween and 1% dry milk in PBS. Mouse mAbs recognized P0 (P07, the generous gift of Dr. Juan Archelos, Department of Neurology, Karl-Franzens-Universitat, Graz, Austria; Archelos et al. 1993) and ß-tubulin (Sigma Chemical Corporation, St. Louis, MO, US). Peroxidase-conjugated secondary antibodies (Sigma Chemical Corporation, St. Louis, MO, US) were visualized using the ECL method with autoradiography film (Amersham Biosciences AB, Uppsala, Sweden). The intensity of bands was quantified by densitometry, and the ratio of intensities for each myelin protein and ß-tubulin was determined. Antibodies: chicken anti P0 (Aves); mouse anti ß-tubulin (Sigma); rabbit anti Calnexin (Sigma).

***Analysis of MPZ expression in patient material***

A skin biopsy was taken after informed consent from a patient carrying a de novo Q215X mutation and a parent as non-carrier control. The case was described in Shy et al (Brain, 2004) using the previous MPZ nomenclature and therefore referred to as Q186X. We extracted RNA from the biopsies, performed RT-PCR and used Fnu4HI enzyme digestion to discriminate the allele carrying the Q215X mutation, which becomes insensitive to digestion, from the wild-type counterpart.

***Semi-quantitative RT-PCR***

Sciatic nerves were dissected from mutant and control littermates at the ages indicated. Total RNA was isolated using the triazol reagents (Boehringer Mannheim, Mannheim, Germany) with minor modification. Nerves were homogenized in the presence of triazol, extracted with chloroform and precipitated in the presence of tRNA. A portion (500 ng) of total RNA was reverse transcribed using Moloney Murine Leukaemia Virus reverse transcriptase and oligo dT primers (Promega Corporation, Madison, WI, US). For analysis in sciatic nerves, equal volumes of the reverse-transcribed product from nerves of mutant and control mice were amplified using GAPDH-specific primers (5'-GTATGACTCTACCCACGG-3' and 5'-GTTCAGCTCTGGGATGAC-3') in the presence of alpha 32P-dCTP. PCR conditions were 95°C for 30s, 55°C for 60s and 72°C for 60s (30 cycles), followed by 10 min extension at 72°C, in a standard PCR reaction mix. Aliquots from the amplification were withdrawn at 22, 24 and 26 cycles, resolved on an acrylamide gel and visualized by autoradiography. The intensity of the bands was quantified by densitometry (Molecular Dynamics), to verify that amplification was logarithmic, and to determine the relative amount of starting cDNA from each sample. Equal amounts of RT product, as determined by the GAPDH signal, were amplified using P0 specific primers. Analysis of the products was conducted as for GAPDH. To analyse P0 expression in LoxP and wild type mice, we exploited the exon 3 polymorphism using the RT-PCR method as described in Wrabetz et al. (2000). Briefly, 200 ng of total RNA was reverse transcribed, PCR amplification was performed as described above in the presence of alpha 32P-dCTP, using a single primer pair recognizing P0 exon 2 (5'-GTCCAGTGAATGGGTCTCAG-3') and exon 4 (5'-GCTCCCAACACCACCCCATA-3') that flank a polymorphic DpnII site present only in the C57BL/6J allele and not in 129SVPas (the polymorphic BglII site spans the intron 2/exon 3 boundary, such that in the cDNA product, only its 4 nucleotide core, a DpnII site, remains). PCR conditions were 95°C for 30s, 63°C for 60s and 72°C for 60s (26 cycles), followed by 10 min extension at 72°C, in a standard PCR reaction mix. To avoid the formation of heteroduplexes between the DpnII-containing and non-DpnII-containing products, only cycles in the logarithmic range were chosen. Two microlitres of RT-PCR product were digested with DpnII for 60 min, phenol extracted, precipitated, resolved by acrylamide gel electrophoresis and visualized by autoradiography. The intensity of the bands was quantified by densitometry, and the ratio between DpnII-containing (from wild type mice) and non-DpnII-containing (from LoxP mice) products was calculated. To analyse P0 expression in Q215X mice, we took advantage of the BsoFI site that disappear when the C to T transition in exon 5 is present. We used the RT-PCR method of Fiering et al (1995) described above. We performed PCR amplification using a P0 specific primer pair that flank the Q215X mutation in exon 5 (in exon 4: 5’-GGCAGGCTGCCCTGCAG-3’ and in exon 6: 5’-CTTCTCACTGGCAGCTTTGGTGC-3’), in the presence of 32P-dCTP. PCR conditions were 95°C for 30s, 63°C for 30s and 72°C for 30s (26 cycles), followed by 7 min extension at 72°C, in a standard PCR reaction mix. The RT-PCR products were digested with BsoFI for 60 min, phenol extracted, precipitated, resolved by acrylamide gel electrophoresis and visualized by autoradiography. The intensity of the bands was quantified by densitometry, and the ratio between BsoFI-containing (from wild type Mpz allele) and non-BsoFI-containing (from Q215X allele) products was calculated.

***Semi-quantitative and quantitative Real Time-PCR***

Sciatic nerves from WT and transgenic mice were immediately frozen in liquid nitrogen after dissection. Total RNA was prepared with Trizol (Roche Diagnostic GmbH, Germany), and 1 μg of RNA was reverse transcribed using 1 mM dNTPs, 2.5 ng/ml random examers, 40 units RNasin, and SuperScriptII RNase H- Reverse Transcriptase (Invitrogen, U.S.A.) as per manufacturer's instructions. Quantitative PCR was performed according to the manufacturer’s instructions (Taqman, PE Applied Biosystems Instruments) on an ABI PRISM 7700 sequence detection system (Applied Biosystems Instruments). The relative standard curve method was applied using WT mice as reference. Normalization was performed using either 18S rRNA or phosphoglycerate 1 (PGK1) as reference genes. Target and reference gene PCR ampliﬁcation were performed in separate tubes with Assays on Demand (Applied Biosystems Instruments). Total RNA was prepared as described above from normal and mutant mice. To measure each gene, 1 μg of RNA was retrotranscribed as described above. Assay on Demand™ (Applied Biosystems Instruments): 18S assay Hs99999901_s1; PGK1 assay Mm00435617_m1; XBP Mm00457357_m1; BIP assay Mm00517691_m1.

***Nonsense mediated decay (NMD)***

In order to test whether Mpz transcripts undergo nonsense mediated decay in the mouse sciatic nerve, we treated excised and desheathed SNs with cycloheximide (CHX) in culture medium for 6 hours. After CHX treatment, mRNA was extracted and retro-transcribed and expression was evaluated by RT-PCR.

***Immunoprecipitation***

After lysis, sciatic nerves extract has been incubated with a rabbit anti myc (Upstate) antibody for 2 hours at 4°C on a rotating wheel. The lysate was incubated with sepharose-protein A (GE Healthcare) for 2 hours at 4°C. After washing with lysis buffer plus detergent and lysis buffer without detergent, elution of immunoprecipitated samples was performed by boiling beads for 10 minutes in DTT containing sample buffer. Aliquots of the lysate or the first wash (void) were collected and resolved by Western Blot alongside immunoprecipitation samples.

***Morphological Analysis***

Mutant and control littermates were sacrificed at the ages indicated and sciatic nerves were dissected. In most cases, semi-thin section and electron microscope analyses of sciatic nerves was performed as described in Quattrini et al., 1996. For semi-thin analysis, a portion of the nerve was fixed in 2% buffered glutaraldehyde and postfixed in 1% osmium tetroxide. After alcohol dehydration, these samples were embedded in Epon. Transverse sections (0.5-1 μm thick) were stained with toluidine blue and examined by light microscopy. We analysed 5 mice per genotype and we took 4-5 images at 100x magnification. A minimum of 30 fibres per image were counted per genotype. Ultra-thin sections were stained with uranile acetate and lead citrate and examined by electron microscopy. Axonal diameter in electromicrograph was determined using the NIH ImageJ software (HYPERLINK "http://rsb.info.nih.gov/ij/"http://rsb.info.nih.gov/ij/). A minimum of 15 images were counted per genotype (3 mice/genotype).

***Behavioral Analysis***

For Rotarod analysis, groups of P10 old mutant and control littermates were placed on a round metal bar rotating first at 4 rotations per minute (rpm) and then accelerating at 7.2 rpm2 (Ugo Basile, VA, Italy). The animals were allowed to stay on the rod for a maximum of 900 s and the time of hold on the rotating rod was measured in subsequent trials (2 trials per day from P10 to P12). Statistics was made using the SigmaStat 3.0 software.

For grid walking test, the animal is placed on two-overlapped grids forming a 1cm x 1cm mesh. Grid test consist on one-day habituation followed by one-day test and animals are free to walk for two minutes. We calculated the number of times in which the animal’s hind paw falls into a mesh of the grid (number of error) and total number of steps.

***Teasing on sciatic nerves and Immunohistochemistry analysis***

Single fibers of P28 sciatic nerves from transgenic and control mice were teased apart after removal of the perineurium using fine stainless-steel needles, dried, and stored at -20°C. Teased fibers were obtained after a 30-minute 4% paraformaldehyde fixation of the whole nerve, subsequently kept in PBS 1X at 4 °C until teasing. Slides with teased fibers were fixed in 4% paraformaldehyde for 10 minutes at room temperature and, when indicated, followed by immersion in cold methanol for 10 minutes and two rinses in PBS 1X. Specimens were blocked in 0.1% Triton X-100 and 10% normal goat serum (Dako, Glostrup, Denmark) in PBS 1X for 1h at room temperature. Primary antibody incubation was 1 h or more at RT using chicken anti-P0 (1:300, Aves) and KDEL antibody (Stressgene). Slides were then rinsed in PBS 1X and incubated for 1 h with FITC- conjugated secondary antibody (The Jackson Laboratory, Baltimore, MD), stained with DAPI, mounted with Vectashield (Vector Laboratories, Burlingame, CA), and examined on a confocal microscope (Ultraview). Image analysis was performed with ImageJ software as follows. From teased fibers slides, images of myelinating Schwann cell nuclei disposed on the side of the axon were acquired to avoid an overlay with the underlying myelin. A perinuclear triangular area was selected as mask using the green and blue channel image and P0 fluorescence average intensity was measured in the red channel image, always verifying that the range was never over the saturation threshold.

***Image Analysis***

Micrographs of morphological samples and radiographic films were digitalized using an AGFA Arcus 2 scanner and figures were prepared using Adobe ® Photoshop 7.0.

***Immuno-electronmicroscopy***

Transgenic and wild type mice at P10 were perfused with 4% paraformaldehyde/2.5% glutaraldehyde in 0.08 M sodium phosphate buffer (pH7.3). Sciatic nerves were excised and placed in fixative overnight at 4°C. Nerves were maintained in increasing gradients (1M, 1.5M, 2M, 2.3M) of sucrose-polyvynilpyrrolidone (PVP) solution for 24 hours each incubation and then stored at 4°C in 2.3M sucrose/30% PVP for three days before cutting. Tissues were cut in little pieces and frozen in liquid nitrogen. Specimens were then cut in ultrathin cryosections (≤ 60 nm thick) with an Ultracut UCT Ultramicrotome (Leica Instruments) and placed on nickel formvar coated grids. The sections were processed as described previously (Trapp et al., 1989, Yin et al., 2000) with the following modifications. The grids were left in a 2.3M sucrose solution overnight at 4°C before proceeding with immunostaining. Specimens were treated with gelatin 2% 2x5min and PGB (0.1M glycine, 1% BSA, in PBS 1X) 2x5min, and incubated with primary antibody (chicken P0, Aves, 1:600 in PGB) for 1 hour at 37°C. Sections were then rinsed in PGB and incubated with secondary antibody (1:200 rabbit anti chicken – 10nm gold particles conjugated) for 1 hour at room temperature. After rinsing in PBS1X and in cacodylate buffer 0,12M, tissues were stained in osmium 1% in cacodylate buffer 0,12M for 5min and then in saturated uranyl acetate for 15min. Tissues were then rinsed in water, dehydrated in ethanol 20%-50%-70%-95%-100% and finally incubated in ethanol 100%-LR White 1:1 and then in LR white quickly, and the grids were left overnight at 60°C. The grids were then stained in saturated uranyl acetate and lead citrate and finally examined by EM. A minimum of 40 images were analysed per genotype (3 mice/genotype); these experiments were repeated twice on different sets of animals.

***Confocal and electron microscopy***

Images of teased fibers were taken at the PerkinElmer UltraVIEW ERS Spinning Disk confocal microscope; images of IEM were taken at the Trasmission Electron Microscope Leo 912AB; MDCK images were taken at Leica TCS SP5 II confocal microscope.

**Engineering and Cloning**

pCDNA3.1(+) (Invitrogen) is a 5.4Kb vector used for the expression of protein in eukaryotic cells under the CMV promoter. Downstream the CMV promoter, a Multicloning Site and a Bovin Growth Hormone– BGH polyadenilation sequence are present. A SV40 replication origin for expression in mammalian cells and a pUC replication origin for expression in E.Coli are present. This vector has an amplicillin resistance cassette. pBS-HA vector is a 3.4 Kb vector for prokaryotic expression. The HA fragment has been inserted in the middle of a multicloning site. This vector has an amplicillin resistance cassette. pcDNA3.1(+) P0HA. Rat P0 cDNA has been cloned HindIII/BamHI in pBS-HA vector. Then the P0-HA fragment has been inserted in a pcDNA3.1(+) vector HindIII/EcoRV. The P0HA fragment has been modified via PCR-based mutagenesis (primer P0BamHI-F and P0BamHI-R see below) to have the correct frame between P0 and HA: deletion of two basis after the last codon of P0 was required. Primer used: P0HABamH1-F 5’- CGCAAGGATAAGAAAGGATCCCCCGGG -3’; P0HABamH1-R 5’- CCCGGGGGATCCTTTCTTATCCTTGCG- 3’. pcDNA3.1(+) Q215XP0HA. The QuikChangeTM Site-Direct Mutagenesis Kit (STRATAGENE) has been used to amplify the P0 cDNA fragment from HindIII site, upstream ATG codon, to 214 P0 codon in pcDNA3.1(+) P0HA and to introduce a BamH1 site at the 3’. In fact, the primers have been designed in order to have HindIII digestion site (primer 186HA-Fw) and BamHI digestion site (primer-186HA Rv) at 5’ and 3’; after mutagenesis, the product has been digested with HindIII and BamHI in order to obtain a HindIII-BamH1 P0 fragment that ends at 214 codon; the HindIII and BamHI digestion sites are necessary to subsequently subclone the fragment in frame with HA tag. To do that, pcDNA3.1(+) P0HA has been cut with HindIII-BamH1 to excise full length P0 and then to insert the 214P0 fragment in frame with HA tag using T4 ligase (Promega) as per manufacturer’s instructions. Primer 186HA-Fw: 5’ GGAGACCCAAGCTGGCTAGCG 3’; primer 186HA-Rv: 5’ CGGGATCCCCGCCCTCGCTTC 3’. pcDNA3.1(+) Q215XP0-YAML-HA. pcDNA3.1(+) Q215XP0HA has a BamH1 site before HA tag and EcoRI site after the HA stop codon. A forward and a reverse primer (primer YAML-HA Fw and YAML-HA Rv) have been designed to have the YAML sequence and the HA tag, in frame, with a BamH1 site at 5’ and an EcoRI at 3’; these primers are complementary, and they have been annealed together (5 minutes at 96°C and then cooled at room temperature) to obtain a stable product. pcDNA3.1(+) Q215XP0HA has been cut with BamHI-EcoRI to excise HA tag; subsequently, the annealed primers have been inserted in this vector to obtain the 214P0 fragment with the YAML sequence and the HA tag in frame. The ligation reaction has been performed using T4 ligase (Promega) as per manufacturer’s instructions. Primer YAML-HA Fw: 5’ GATCCTATGCCATGCTGCCCGGGCTGCAGATGTACCCATACGATGTTCCAGATTACGCTAGCTTGTGATAAGG 3’; primerYAML-HA Rv: 5’ AATTCCTTATCACAAGCTAGCGTAATCTGGAACATCGTATGGGTACATCTGCAGCCCGGGCAGCATGGCATAG 3’. Both pcDNA3.1(+) Q215XP0HA and pcDNA3.1(+) Q215XP0-YAML-HA constructs have been sequenced to be confirmed.

***Cell lines***

MDCK (ATCC) cell line has been cultured in E-MEM, 10% FCS, 1% Pen/strep and grown at 37°C, 5% CO2.

***Transfection***

MDCK cells were plated on coverslips in a 6-wells plate at 90% of confluence, and transiently transfected using Lipofectamine 2000 reagent (Invitrogen), as per manufacturer's instructions. 4 μg of DNA was transfected for immunocytochemistry.

***Immunocytochemistry***

48 hours after transfection cells have been fixed with PFA 4% for 20 minutes, washed twice with PBS 1X and permeabilized with triton 0.5%. After washing, the cells were incubated in blocking solution (3% BSA) for 1-hour room temperature and then incubated over-night with primary antibody: rat anti HA 1:100 (Roche), mouse anti ZO1 fitc-conjugated 1:300 (Invitrogen), mouse anti Beta-Catenin 1:300 (BD Transduction Laboratories). After washing, cells have been incubated with secondary antibody, with DAPI and mounted with Vectashield (Vector Labs, Baltimore, PA, U.S.A.). The cells were imaged with an Ultraview confocal microscope. Cells were imaged in the XZ plane, though some areas were images in the XY plane or as XYZ series.

***
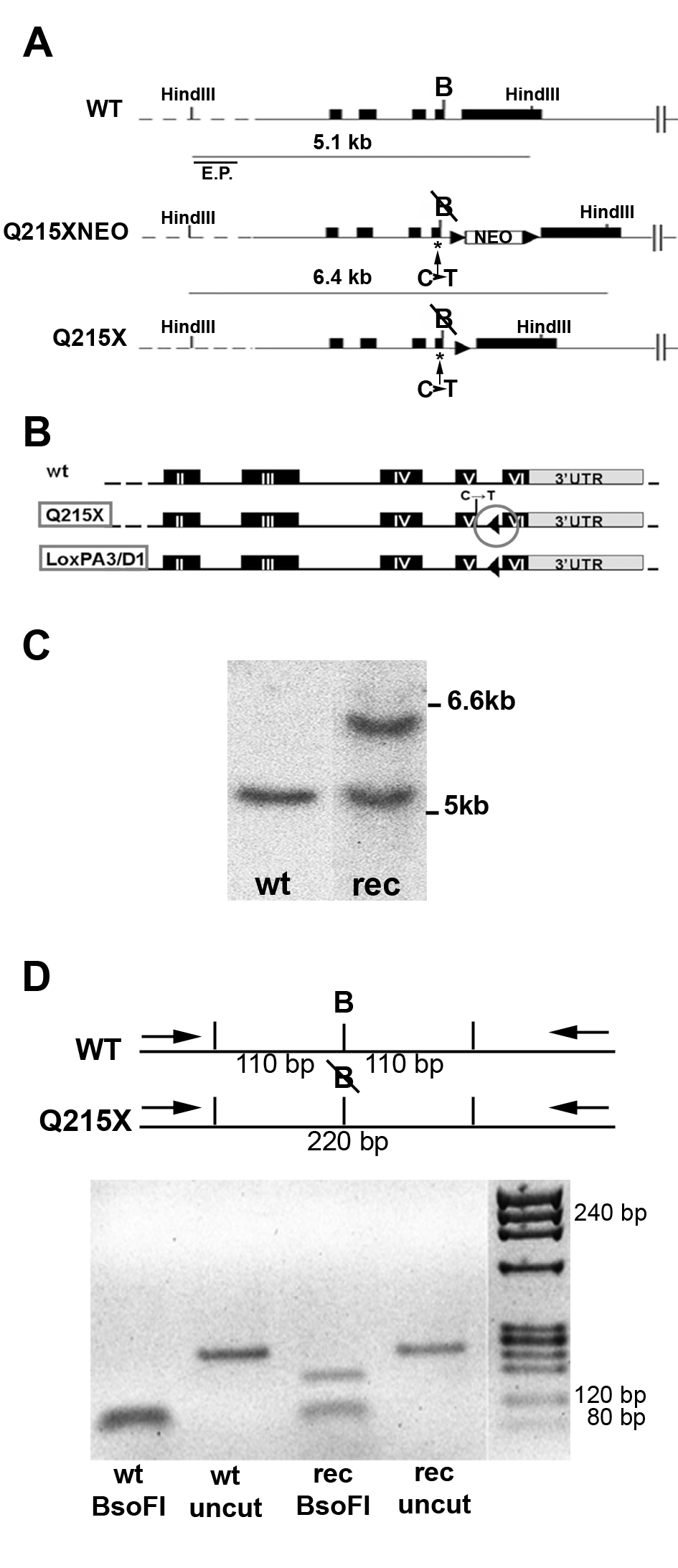
Supplementary figure 1. Generation of Q215X mouse line.***

**A.** Schematic representation of the genomic organization of wild-type, Q215XNEO and Q215X *Mpz* alleles. The external probe used for Southern blot analyses of ES cell clones is indicated (E.P.), together with the length of the DNA fragments, originating upon HindIII digestion. The C to T mutation in exon 5 is indicated by asterisks. The BsoFI site within exon 5 is indicated (B). **B.** Structure of P0Q215X and LoxP gene with the LoxP site (circle) in the intron 5. **C.** Southern Blot analysis of the genomic DNA of the ES cell clone where homologous recombination occurred. **D.** BsoFI restriction enzyme digestion of the PCR-amplified genomic region flanking the C to T mutation in exon 5. The BsoFI site within exon 5 is indicated (B).

***
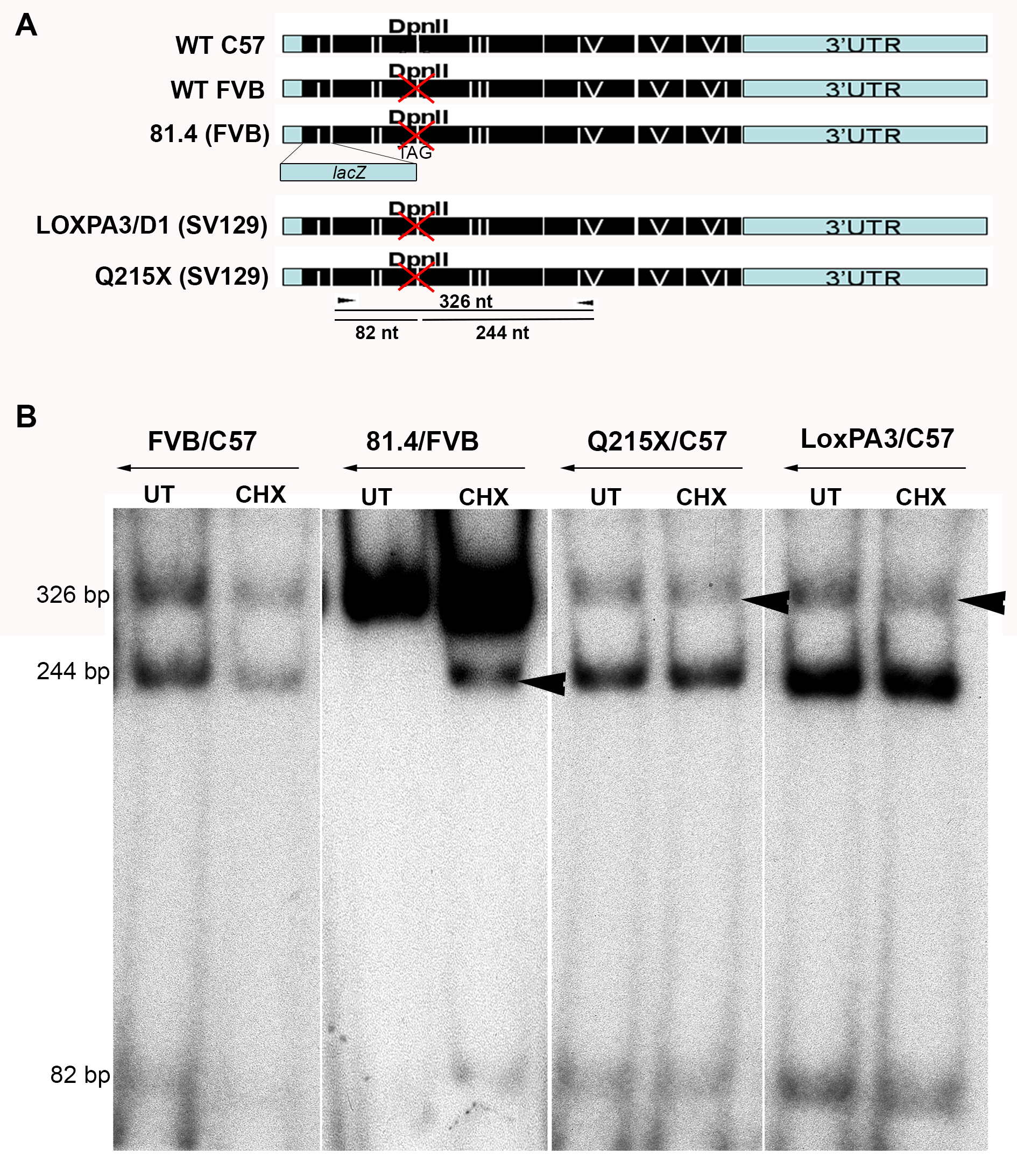
***

***Supplementary figure 2. Q215X mutation and intron 5 LoxP sites do not induce nonsense mediated decay (NMD).***

**A.** Schematic representation of the RT-PCR primers and DpnII restriction enzyme sites in the different *Mpz* alleles. Mpz^tg81.4^, an *Mpz* allele containing lac-z and a premature stop codon, is predicted to undergo NMD and was used as a positive control (Feltri et al, 1999). **B.** *Mpz* allele sensitivity to NMD was tested by comparing Mpz levels in untreated (UT) SNs with ones treated with cycloheximide (CHX), which inhibits NMD. No difference in Q215X and LoxP-WT transcript expression was detected after treatment, while, as expected, there was a significant increase in the Mpz^tg81.4^ transcript (positive control). These results confirm that in an *in vivo* in a myelin context, Q215X mutation and intron 5 LoxP sites do not induce NMD.

***
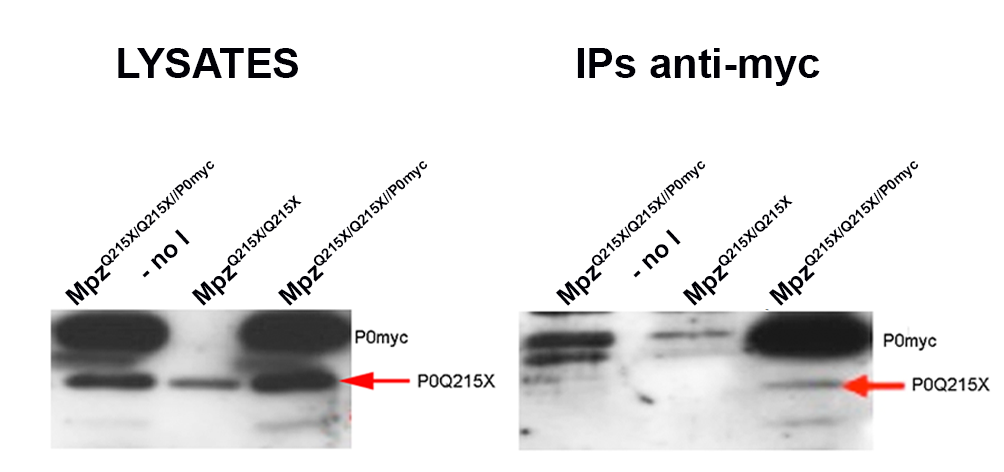
***

***Supplementary figure 3. Interaction between P0Q215X and WTP0.***

Immunoprecipitation for P0myc in *Mpz^Q215X/Q215X^*^//P0myc^ mice. The red arrow indicates the bands corresponding to the truncated P0Q215X in the lysates and in the IP. Anti-myc antibodies are able to co-IP P0myc and P0Q215X from *Mpz^Q215X/Q215X^*^//P0myc^ mice. As negative controls, sciatic nerve lysates from *Mpz^Q215X/Q215X^*^//P0myc^ mice were exposed to only beads (no myc antibody, (no I)) or sciatic nerve lysates from *Mpz^Q215X/Q215X^* were immunoprecipitated with myc antibody. In these two lanes there are no bands corresponding to P0Q215X, demonstrating that P0Q215X is not simply adhering directly to the beads.
